# Importance of Translabial Ultrasound for the Diagnosis of Pelvic Organ Prolapse and Its Correlation with the POP-Q Examination: Analysis of 363 Cases

**DOI:** 10.3390/jcm10184267

**Published:** 2021-09-20

**Authors:** Gina Nam, Sa-Ra Lee, Sung-Hoon Kim, Hee-Dong Chae

**Affiliations:** 1 Department of Obstetrics and Gynecology, Chung-Ang University Hospital, Chung-Ang University College of Medicine, 102, Heukseok-ro, Dongjak-gu, Seoul 06973, Korea; ginanam@caumc.or.kr; 2 Department of Obstetrics and Gynecology, Asan Medical Center, University of Ulsan College of Medicine, 88, Olympic-ro 43-gil, Songpa-gu, Seoul 05505, Korea; kimsung@amc.seoul.kr (S.-H.K.); hdchae@amc.seoul.kr (H.-D.C.)

**Keywords:** enterocele, pelvic organ prolapse, pelvic organ prolapse quantification (POP-Q), rectocele, translabial ultrasound

## Abstract

The incidence of pelvic organ prolapse (POP) is increasing in our aging society. We aimed to evaluate the clinical usefulness of translabial ultrasound (TLUS) by comparing the findings of POP-Q examination and TLUS in advanced POP patients and we also aimed to evaluate the prevalence of rectocele and enterocele on the TLUS. We analyzed the TLUS and POP-Q exam findings of 363 symptomatic POP patients who visited our clinic from March 2019 to April 2021. We excluded three patients who had conditions mimicking POP, as revealed by the TLUS. The most common POP type was anterior compartment POP (68.61%), followed by apical compartment (38.61%) and posterior compartment (16.11%) POP. Agreement between the POP-Q exam and TLUS was tested using Cohen’s kappa (κ). *p* values < 0.05 were considered statistically significant. The incidence of rectocele or enterocele was only 1.67% (6/360) and there was no rectocele or enterocele in most patients (246/252, 96.63%) when the POP-Q exam revealed posterior compartment POP, suggesting that they only had posterior vaginal wall relaxation. The positive predictive value of the POP-Q exam for detecting rectocele or enterocele (as revealed by TLUS) was only 2.38%, whereas the negative predictive value was 100%. In conclusion, the application of TLUS is useful in the diagnosis of POP, especially for differentiation of true POP from conditions mimicking POP. The correlation between the POP-Q exam and TLUS is low, especially in posterior compartment POP, and therefore, patients with POP-Q exam findings suggesting posterior compartment POP should undergo TLUS to check for rectocele or enterocele. The use of TLUS in the diagnosis of POP patients can improve the accuracy of the diagnosis of POP patients in conjunction with a POP-Q exam.

## 1. Introduction

Pelvic organ prolapse (POP) is a condition in which the pelvic organs such as the bladder, uterus, rectum, and sometimes the small bowel descend from their normal position through the vagina. The prevalence of POP is higher in older women, with a lifetime risk factor of 30% and 180 per 100,000 women aged 50 or older based on a database maintained by the Korea National Health Insurance [1]. Symptoms of POP include a sensation of vaginal bulge, overflow urinary incontinence, urinary retention, voiding difficulty, constipation, and splint behavior during urination or defecation [2]. Aging and parity are independent risk factors for POP [3]. The lifetime risk of surgery for POP in women is 19% [4]. The surgical repair of POP is the most common inpatient procedure performed in women aged 70 or older [5] and 1012 million USD is estimated as the economic burden of POP in our aging society [6].

**Figure 1:**
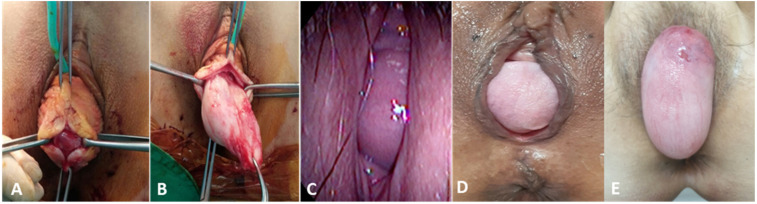
Conditions mimicking pelvic organ prolapse (POP) and true POP. (**A**,**B**) anterior vaginal wall leiomyoma mimicking anterior compartment POP. (**C**) Urethral diverticulum mimicking anterior compartment POP. (**D**) Rectocele and (**E**) enterocele revealed by translabial ultrasonography.

The POP-Q system of classification proposed by the International Incontinence Society (ICS) is used for staging the severity of POP [2]. At present, the standard POP-Q examination provides quantification of all parameters. There are two anterior points (Aa and Ba), two posterior points (Ap and Bp), two apical points (C and D), total vaginal length (tvl), genital hiatus (gh), and perineal body (pb) that need to be determined. In terms of POP-Q staging, the 0 to IV stage is determined depending on the distance of the most dependent portion of the pelvic organ from the hymenal ring [7]. This system is a standardized method in widespread use and has excellent inter-observer and intra-observer reliability [3]. However, this is still a relatively subjective measurement; it can be affected not only by the examiner’s skill but also the patient’s ability to perform the maximal Valsalva maneuver at the time of the POP-Q exam. In addition, we cannot exactly know which organ is in the prolapsed vaginal pouch, and there can also be both pelvic organs and/or a mass. Many conditions mimicking POP, sometimes called false POP, have been reported [8,9,10] (Figure 1).

Furthermore, even in cases of true POP, especially in posterior compartment prolapse, the presence of rectocele or enterocele cannot be clearly determined only by physical examination, including POP-Q staging. In cases of complete uterovaginal prolapse, most examiners think that the rectum or small bowel is in the pouch of the posterior vaginal wall, the fully prolapsed state. However, we often encounter only a relaxed posterior vaginal wall forming a pouch that does not contain the rectum (rectocele) or small bowel (enterocele) (Figure 1). Treatment also needs to be individualized according to the anatomical situation and the presence of rectocele or enterocele. A defect of the rectovaginal septum should be closed in rectocele, perineal hypermobility is cured by levatorplasty, and a deficient perineum requires a perineoplasty [11]. The differential diagnosis of the presence of true rectocele or simple posterior vaginal relaxation is easy with TLUS. Although some authors concluded that, for asymptomatic rectoceles, a concurrent posterior repair at the time of surgical repair of apical prolapse reduces the risk of surgical failure, there is still debate on it [12,13]. The need for routine posterior repair should be reevaluated after the differential diagnosis of the presence of true rectocele or simple posterior vaginal relaxation to reduce unnecessary posterior repair for simple vaginal relaxation. In addition, the surgical treatment of POP with enterocele should include the enterocele repair and the removal of the enterocele sac. Repair of rectovaginal septum without enterocele repair in this case results in POP recurrence.

Therefore, a need for imaging techniques for the evaluation of POP has become apparent. Traditionally, defecography has been used for evaluating posterior compartment prolapse with rectocele and enterocele. Since the early 2000s, translabial ultrasound (TLUS) and dynamic magnetic resonance imaging (MRI) with numerous modification methods have been used to assess the genitourinary tract and the degree of POP [4,5,6,7]. While defecography is relatively unpleasant and MRI is costly, TLUS is a simple, inexpensive, and non-harmful diagnostic modality that is easy to perform and widely applicable in most gynecologic clinics. However, there are only a few reports on the relationship between clinical symptoms, POP-Q stage, and TLUS findings [11,14,15,16,17,18].

Therefore, we aimed to evaluate the clinical usefulness of TLUS by comparing the findings of POP-Q examination and TLUS in advanced POP patients. Furthermore, we also aimed to evaluate the prevalence of rectocele and enterocele on TLUS and the main POP type according to the age distribution in Korean POP women.

## 2. Materials and Methods

**Figure 2:**
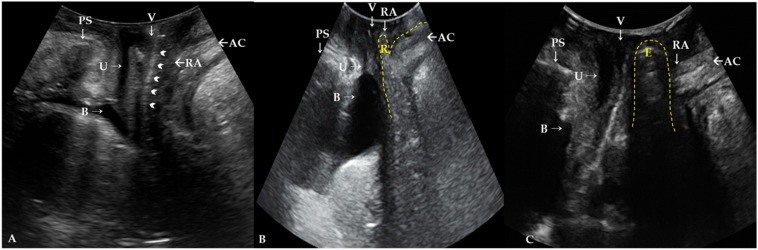
Translabial ultrasonography (**A**) in resting phase. Rectovaginal septum (arrowheads). AC, anal canal; B, bladder; PS, pubis symphysis; RA, rectal ampulla; U, urethra; V, vagina. Translabial ultrasonography in patients with (**B**) rectocele and (**C**) enterocele in maximal Valsalva phase. Rectocele filled with stool and air, resulting in hyperechogenicity, and the defect of the rectovaginal septum is observed in the maximal Valsalva maneuver (**B**). The contents of an enterocele appear generally iso- to hyperechogenic compared with a rectocele and bowel peristalsis is usually observed in the enterocele sac (**C**). R, rectocele; E, enterocele.

This is a retrospective review of a total of 363 female patients who visited our outpatient clinic from March 2019 to April 2021 for a protruding vaginal mass suspicious of POP. On physical examination, the patients were routinely assessed by a single urogynecologist (Dr. L.S.R. with over 15 years of experience) using the ICS POP-Q exam. The measured parameters were Ba, C, Bp, gh, and pb as defined by the ICS POP-Q [7]. Each patient’s main prolapsed compartment was defined as the most dependent compartment among the prolapsed compartments and designated as the anterior, apical, or posterior compartment, represented by Ba, C, and Bp on the POP-Q exam, respectively. TLUS was also routinely assessed by the urogynecologist (Dr. L.S.R.). A transabdominal probe, a 3.5–5 MHz convex array transducer, was applied on the labium majora with the patient in the semi-Fowler position after voiding before the exam. Even after the full voiding, some patients were in the bladder-filling state for the incomplete voiding for the cystocele. Measurements of TLUS are performed at rest and during a maximal Valsalva maneuver. The presence of rectocele or enterocele were defined as the descent of either the rectal ampulla or the small bowel, sigmoid colon, or omentum into the Douglas pouch as defined in the previous literature [11]. The peritoneal cul-de-sac filled with echogenic small bowel and peristalsis was obvious in cases of enterocele during a maximal Valsalva maneuver. Examples of TLUS at rest and during a maximal Valsalva maneuver in patients with rectocele, enterocele, and procidentia without rectocele or enterocele, with just posterior vaginal wall relaxation, are shown in Figure 2.

Conditions mimicking POP, a false POP, such as urethral diverticulum, leiomyoma, vaginal cyst, or hemangioma, were also assessed during TLUS. A total of three patients, one patient with urethral diverticulum, one patient with an epidermoid cyst, and one patient with an anterior vaginal leiomyoma, were excluded from this study.

### Statistical Analysis

To assess the association between normally distributed continuous variables, we used Pearson’s correlation test. Very weak, weak, moderate, and strong correlations were defined as r of 0 to 0.1, 0.1 to 0.3, 0.3 to 0.7, and 0.7 to 1.0. The strength of the association between normally distributed continuous variables and ordinal variables was determined by Spearman correlation coefficients. Very weak, weak, moderate, and strong correlations were defined as rs of 0 to 0.1, 0.1 to 0.3, 0.3 to 0.7, and 0.7 to 1.0. Agreement between clinical definition of posterior compartment POP using POP-Q and TLUS was tested using Cohen’s kappa (κ). Poor, fair, moderate, good, and very good agreement were defined as κ of <0.2, 0.21 to 0.40, 0.41 to 0.60, 0.61 to 0.80, and 0.81 to 1.00. *p* values < 0.05 were considered statistically significant. The statistical analysis was performed using SPSS for Windows version 20 (SPSS Inc., Chicago, IL, USA).

## 3. Results

Among 363 patients who visited our outpatient clinic due to a suspicion of POP, a total of 360 POP patients who were diagnosed as true POP by the POP-Q exam and TLUS were included in the final analysis. A total of 41 (11.39%) patients were vault prolapse patients with a history of hysterectomy. All parameters were normally distributed on Kolmogorov–Smirnov testing. The age distribution of the patients is summarized in Table 1. The mean age ± standard deviation of the participants was 59.34 ± 13.22 (range, 29–87) years. Among the patients, the largest proportion (125 (34.72%)) were in their 60s.

The mean POP-Q points, stage, and main compartment of prolapse in our study population are listed in Table 2. On clinical examination, 360 women had POP of ICS stage 2 or higher. The main compartment of prolapse was distributed as follows: anterior compartment in 247 (68.61%), apical compartment in 139 (38.61%), and posterior compartment in 58 (16.11%).

Table 3 shows the correlation between age and the parameters of the POP-Q measurements. Age was significantly correlated with points Aa (rho = 0.43; *p* < 0.001), Ba (rho = 0.35; *p* < 0.001), Bp (rho = 0.12; *p* = 0.03), and pb (rho = −0.13; *p* = 0.02). Increasing age was also positively correlated with the prolapse stage (rho = 0.24; *p* < 0.001).

The agreement between the clinical definitions using the POP-Q and TLUS for anterior, apical, and posterior compartment prolapses is shown in Table 4. The agreement is poor and not significant in anterior compartment POP (Cohen’s Kappa = 0.01; *p* = 0.17), apical compartment POP (Cohen’s Kappa = 0.02; *p* = 0.54), and posterior compartment POP (Cohen’s Kappa = 0.01; *p* = 0.11). The clinically significant posterior compartment of POP using POP-Q has a positive predictive value (PPV) of 2.38% and a negative predictive value (NPV) of 100%. PPV of posterior compartment prolapse is lower than that of anterior and apical compartment prolapse.

## 4. Discussion

In this study, we demonstrated the usefulness of TLUS by revealing that there is no rectocele or enterocele in most patients (246/252, 96.63%), which is not detectable by the POP-Q exam and suggests posterior compartment POP with Bp point >−3, which can be interpreted to mean that they only had posterior vaginal wall relaxation. The PPV of POP-Q exam for detecting rectocele or enterocele on TLUS was only 2.38%, whereas the NPV was 100%.

The incidence of rectocele or enterocele was only 1.67% (6/360), which is much lower than our expectations based on the previous literature [19]. We also identified three patients who had conditions mimicking POP by using TLUS.

The most common POP type was anterior compartment POP (68.61%), followed by apical compartment (38.61%) and posterior compartment (16.11%) POP. The majority of the women who visited our clinic for symptomatic POP were in their 60s.

### 4.1. Correlation of TLUS Findings and the POP-Q Exam Findings

To date, the main purpose of TLUS has been investigation of cystocele, ureterocele, enterocele, and rectocele, which can be misdiagnosed by the POP-Q system. Dietz et al. [11] first stated that TLUS can be used to quantify POP and that it is correlated with the clinical staging of the POP-Q system. The authors reported that TLUS can demonstrate POP using the inferior margin of the symphysis pubis as a reference line [6]. The maximal descent of the bladder, uterus, cul-de-sac, and rectal ampulla during the Valsalva maneuver was measured [15]. Unlike our study results, Dietz et al. have reported a good correlation for anterior and central compartment prolapses between ultrasound findings and clinical staging [14]. Some authors have reported that the measurement of the anorectal junction position at rest or the anorectal junction movement during straining using TLUS is both simple and accurate [20].

In terms of rectocele, it is assumed to be a result of rectovaginal septal injury during childbirth [21], but it sometimes occurs in nulliparous women. Therefore, it is difficult to distinguish between true and false rectocele and investigate whether a clinically apparent rectocele is due to perineal hypermobility, a defect of the rectovaginal septum, or is an isolated enterocele [11]. True rectocele due to a defect of the rectovaginal septum can also be identified in the mid-sagittal plane on TLUS [5]. TLUS reveals it as a herniation of the rectal wall with a discontinuity in the anterior wall of the anorectum. The depth and width of the herniation during the maximal Valsalva maneuver demonstrate the severity or degree of the rectocele [11]. In our study, the POP-Q system revealed a posterior compartment prolapse, but it was only a relaxation of the posterior vaginal wall with no rectocele or enterocele, which was obvious on TLUS.

### 4.2. Role of TLUS in Differentiation of the True POP from Conditions Mimicking POP

Many conditions, including various vaginal masses such as urethral diverticulum, vaginal leiomyoma, or urogenital cysts, can be confusing factors that mimic POP. A patient with a protruding vaginal mass suggestive of an anterior compartment prolapse with symptoms of urinary frequency and dyspareunia was revealed to have a urethral diverticulum on TLUS [10]. Braga et al. reported a patient who complained of vaginal bulging and overactive bladder symptoms with POP-Q stage II anterior vaginal prolapse (Ba:0), and the histopathological examination revealed an anterior vaginal leiomyoma [22]. Vulvovaginal hemangioma in Klippel–Trénaunay syndrome, vaginal wall cysts, and a perineal mass or even a long cervical polyps can be misdiagnosed as POP [8,9] which manifests the same as our case shown in Figure 1.

Thus, imaging techniques have been studied to identify the defects of the pelvic floor and the relationship with the adjacent organs as a complement to the POP-Q system. The defecation proctography has been regarded as a gold standard in conjunction with a physical examination in assisting diagnostic and surgical approaches to rectocele [23]. MRI has also been introduced for pelvic floor disorders with high-resolution images of the muscles, ligaments, and sphincters, as well as functional information obtained through dynamic MRI [24,25]. However, defecation proctography and MRI are rarely used in the daily clinic because these modalities are relatively expensive, are not very acceptable by the patients, and have risks of radiation exposure or allergic reactions to the contrast media used during the imaging studies. TLUS can be more widely used since ultrasonography is the most widely available user-friendly imaging modality in gynecologic clinics, it is less expensive than MRI, it is easy to perform, it requires only a short time (approximately 5 min), and it can give information as the other two modalities. The urogynecologist (L.S.R.) has been performing surgical treatments for POP patients without performing these two more invasive imaging modalities. Considering the clinical practice in our institute, preoperative defecography is rarely performed in patients who exhibit symptoms or signs of posterior compartment prolapse, even for those who undergo POP operations. Since 2019, the preoperative imaging findings have been evaluated using the TLUS instead of defecography in our institute. We do not perform posterior colpoperineorrhaphy as a routine concurrent procedure for patients with apical POP without symptoms or evidence of rectocele or enterocele on TLUS. Even with this practice pattern, we did not observe a higher de novo rectocele or enterocele after the apical POP operations in this case compared with that reported in the literature [26]. Therefore, the use of TLUS during the diagnosis of POP patients can improve the accuracy of their diagnosis combined with a POP-Q exam with minimal additional effort and cost.

### 4.3. Strengths and Limitations of this Study

This study has several strengths. First, to the best of our knowledge, this is the first study to evaluate the accuracy of TLUS for Korean POP women with a relatively large number of patients. Second, the POP-Q exam and TLUS were performed by a single experienced urogynecologist in all cases over a relatively short period of 25 months. This can exclude interpersonal bias and minimize inter-observer reliability, which can affect accuracy and consistency depending on the experience of the examiner. Finally, although this is a retrospective study design, the data were prospectively collected using the same techniques during routine urogynecologic clinical exams.

However, this study also has some limitations that should be considered. First, this is a single-center retrospective study and not a randomized controlled trial, and it includes no data on women without POP. Second, the degree of rectocele, demonstrated with the depth and width of the rectocele on TLUS, was not analyzed in terms of the stage of POP-Q or Bp point measurement. The severity of POP was not analyzed by quantification of the TLUS using the inferior margin of the symphysis pubis as a reference line, as in the study of Dietz [14]. Only the presence of rectocele or enterocele was analyzed without assessing the burden of symptoms in relation to the TLUS findings. Third, all POP women during the study periods were included, regardless of the operation, because the aim of this study was to compare the POP-Q examination with the TLUS. Therefore, women who did not undergo operations were also included in this study. Finally, this is a single urogynecologist’s experience in a tertiary hospital and we cannot exclude the possibility of selection bias for the analysis of patients who were referred to a tertiary hospital. Therefore, the nationwide Korean statistics cannot be represented with this data, especially for the main type and age distribution.

## 5. Conclusions

In conclusion, the application of TLUS is useful in the diagnosis of POP for the differentiation of true POP from conditions mimicking POP. The correlation between the POP-Q exam and TLUS is low, especially for posterior compartment POP, and therefore patients with POP-Q exam findings suggesting posterior compartment POP should undergo TLUS to check for the presence of rectocele or enterocele.

## Figures and Tables

**Table 1:** Age distribution of 360 women who were diagnosed with pelvic organ prolapse.

Age	*n* (%)
20–29	1 (0.27)
30–39	37 (10.27)
40–49	53 (14.72)
50–59	61 (16.94)
60–69	125 (34.72)
70–79	66 (18.33)
80–87	17 (4.72)

**Table 2:** International Continence Society Pelvic Organ Prolapse Quantification (POP-Q) exam findings.

POP-Q Coordinate (*n* = 360)	Mean ± SD	Range
Aa	0.89 ± 2.03	−4 to 5.5
Ba	2.9 ± 2.54	−3 to 10
C	0.63 ± 4.11	−9 to 10
Ap	−1.65 ± 2.52	−4 to 10
Bp	0.23 ± 3.02	−3 to 10
gh	5.21 ± 1.27	−3.5 to 8
pb	3.57 ± 0.93	−3 to 9
POP-Q stage	3.03 ± 0.44	2 to 4
Main compartment of POP on POP-Q exam, *n* (%)		
Anterior	247 (68.61)	
Apical	139 (38.61)	
Posterior	58 (16.11)	

POP-Q, pelvic organ prolapse quantification; Aa, a point on the midline anterior vaginal wall 3 cm proximal to the hymen; Ba, maximum downward displacement of the anterior vaginal wall; Ap, a point on the midline posterior vaginal wall 3 cm proximal to the hymen; Bp, maximum downward displacement of the posterior vaginal wall; C, maximum downward displacement of the cervix or vaginal vault; gh, length of the genital hiatus; pb, length of the perineal body.

**Table 3:** Correlation between age and pelvic organ prolapse quantification (POP-Q) system points.

POP-Q Coordinate	Correlation Coefficient for Age	*p*-Value
Aa	0.43 ^†^	0.00
Ba	0.35 ^†^	0.00
C	−0.03 ^†^	0.58
Ap	−0.01 ^†^	0.92
Bp	0.12 ^†^	0.03
gh	0.06 ^†^	0.24
pb	−0.13 ^†^	0.02
POP-Q stage	0.24 ^‡^	0.00

Aa, a point on the midline anterior vaginal wall 3 cm proximal to the hymen; Ba, maximum downward displacement of the anterior vaginal wall; Ap, a point on the midline posterior vaginal wall 3 cm proximal to the hymen; Bp, maximum downward displacement of the posterior vaginal wall; C, maximum downward displacement of the cervix or vaginal vault; gh, length of genital hiatus; pb, length of perineal body. ^†^ Pearson’s correlation coefficient. ^‡^ Spearman correlation coefficient.

**Table 4:** Agreement between clinical and sonographic measurements based on the findings of pelvic organ prolapse quantification (POP-Q) and translabial ultrasound (TLUS) in 360 patients.

		POP on TLUS		Cohen’s Kappa (*p* Value)	PPV (%)	NPV (%)
		No	Yes			
Anterior compartment POP on POP-Q exam (Ap > −3)	No	16	0	0.01 (*p* = 0.17)	10.76	100
	Yes	307	37			
		Specificity 4.95%	Sensitivity 100%			
Apical compartment POP on POP-Q exam (C > 0)	No	117	20	0.02 (*p* = 0.54)	17.04	85.40
	Yes	185	38			
		Specificity 38.74%	Sensitivity 65.52%			
Posterior compartment POP on POP-Q exam (Bp > −3)	No	108	0	0.01 (*p* = 0.11)	2.38	100
	Yes	246	6			
		Specificity 30.51%	Sensitivity 100%			

The data are given as *n* (%). POP, pelvic organ prolapse; TLUS, translabial ultrasound. PPV, positive predictive value; NPV, negative predictive value.

## Data Availability

The Excel data used to support the findings of this study were supplied by Sa-Ra Lee under license, and requests for access to these data should be made to Sa-Ra Lee, leesr@amc.seoul.kr.
